# Clinical Management of Neuroendocrine Neoplasms in Clinical Practice: A Formal Consensus Exercise

**DOI:** 10.3390/cancers14102501

**Published:** 2022-05-19

**Authors:** Mirco Bartolomei, Alfredo Berruti, Massimo Falconi, Nicola Fazio, Diego Ferone, Secondo Lastoria, Giovanni Pappagallo, Ettore Seregni, Annibale Versari

**Affiliations:** 1 Azienda Ospedaliero-Universitaria di Ferrara, Presidio Ospedaliero Arcispedale Sant’Anna di Cona, 44124 Ferrara, Italy; mirco.bartolomei@unife.it; 2 Department of Medical and Surgical Specialties, Radiological Sciences, and Public Health, Medical Oncology, University of Brescia, ASST Spedali Civili di Brescia, 25123 Brescia, Italy; 3 Pancreas Surgical Unit, ENETS Center of Excellence, San Raffaele Hospital IRCCS, Vita Salute University, 20132 Milan, Italy; falconi.massimo@hsr.it; 4 Division of Gastrointestinal Medical Oncologya and Neuroendocrine Tumors, European Institute of Oncology, 20132 Milan, Italy; nicola.fazio@ieo.it; 5 Endocrinology Unit, Department of Internal Medicine and Medical Specialties, IRCCS, Ospedale Policlinico San Martino, Università di Genova, 16132 Genova, Italy; ferone@unige.it; 6 Nuclear Medicine Unit, Istituto Nazionale Tumori, Fondazione G. Pascale, 80131 Naples, Italy; s.lastoria@istitutotumori.na.it; 7 School of Clinical Methodology IRCCS “Sacred Heart–Don Calabria” Hospital; 37024 Negrar di Valpolicella, Italy; giovanni.pappagallo@gmail.com; 8 Nuclear Medicine Unit, Fondazione IRCCS Istituto Nazionale dei Tumori, 20132 Milano, Italy; ettore.seregni@istitutotumori.mi.it; 9 Nuclear Medicine Unit, Azienda Unità Sanitaria Locale-IRCCS of Reggio Emilia, 42100 Reggio Emilia, Italy; annibale.versari@ausl.re.it

**Keywords:** neuroendocrine neoplasms, management, Delphi, consensus

## Abstract

**Simple Summary:**

Well-structured international guidelines are currently available regarding the management of patients with neuroendocrine neoplasms (NENs). However, in relation to the multiplicity of treatments and the relative rarity and heterogeneity of NENs, there are many controversial issues in which clinical evidence is insufficient and for which expert opinion can be of help. A group of experts selected 14 relevant topics and formulated relative statements concerning controversial issues in several areas on diagnosis, prognosis, therapeutic strategies, and patient follow-up. Specific statements have also been formulated regarding patient management on radioligand therapy (RLT), as well as in the presence of co-morbidities or bone metastases. All the statements were drafted, discussed, modified, and then approved. The Nominal Group Technique (NGT) method was used to obtain consensus. The results of this paper can facilitate the clinical approach of patients with NENs in daily practice in areas where there is scarcity or absence of clinical evidence.

**Abstract:**

Many treatment approaches are now available for neuroendocrine neoplasms (NENs). While several societies have issued guidelines for diagnosis and treatment of NENs, there are still areas of controversy for which there is limited guidance. Expert opinion can thus be of support where firm recommendations are lacking. A group of experts met to formulate 14 statements relative to diagnosis and treatment of NENs and presented herein. The nominal group and estimate-talk-estimate techniques were used. The statements covered a broad range of topics from tools for diagnosis to follow-up, evaluation of response, treatment efficacy, therapeutic sequence, and watchful waiting. Initial prognostic characterization should be based on clinical information as well as histopathological analysis and morphological and functional imaging. It is also crucial to optimize RLT for patients with a NEN starting from accurate characterization of the patient and disease. Follow-up should be patient/tumor tailored with a shared plan about timing and type of imaging procedures to use to avoid safety issues. It is also stressed that patient-reported outcomes should receive greater attention, and that a multidisciplinary approach should be mandatory. Due to the clinical heterogeneity and relative lack of definitive evidence for NENs, personalization of diagnostic–therapeutic work-up is crucial.

## 1. Introduction

Neuroendocrine neoplasms (NENs) are a heterogeneous group of neoplasms that arise from the neuroendocrine cell system [1]. While NENs occur within the gastroenteropancreatic (GEP) system in most cases, they may also arise from other systems. Considering data from a large population-based study, the overall incidence of low/intermediate grade NENs is 25 per 1,000,000 and they appear to be more frequent in patients ≥65 years in whom the incidence reaches 40 per 1,000,000 per year [2]. In addition, there is speculation that the incidence of NENs is increasing, although this may be related to use of more sensitive diagnostic methods and increased awareness among clinicians [1]. The classification system from the International Agency for Research on Cancer (IARC) and World Health Organization (WHO) considers the anatomic location, category, family history, type, and grade of tumor [3]. While well-differentiated NENs are called neuroendocrine tumors (NETs) poorly differentiated NENs are referred to as neuroendocrine carcinomas (NEC). Neuroendocrine neoplasms are clinically classified as functioning or non-functioning, depending on whether the tumor has the ability to secrete biogenic amines or peptide hormones that give rise to clinical symptoms.

A wide range of treatment approaches are now available for NENs, which broadly comprise surgical and ablative treatment, and use of somatostatin analogs (SSAs), targeted agents, chemotherapy, and peptide receptor radionuclide therapy (PRRT)/radioligand therapy (RLT), in addition to watchful waiting in very selected patients [4]. Several societies have issued guidelines for diagnosis and treatment of NENs, including the National Comprehensive Cancer Network (NCCN), European Society for Medical Oncology (ESMO), and European Neuroendocrine Tumor Society (ENETS) [5,6,7]. Notwithstanding, there are still several areas of controversy for which there is limited guidance, and diagnostic and therapeutic protocols may vary significantly among centers according to their expertise and geographic location.

The scope of this paper is to provide a valuable source of guidance where firm recommendations are lacking, made by a group of experts, who discussed current issues and formulated a series of statements relative to diagnosis and treatment, in order to facilitate daily practice in the management of patients with NENs.

## 2. Materials and Methods

The Nominal Group Technique (NGT) is a formal method of obtaining consensus that was developed to overcome a portion of the negative aspects of group dynamics and help ensure that a group decision is made used to obtain consensus [8,9]. The NGT is especially well-suited for obtaining consensus in smaller groups, where extensive face-to-face discussion and exchange of ideas can take place. The NGT is a structured group interaction, and allows participants to express their opinions and have their opinions considered by the other participants, thus overcoming a portion of the negative aspects of group dynamics and help ensure that a group decision is made [8,9]. A maximum of 7 participants is recommended, which is the number of members who took part in the present consensus meeting. The NGT used herein was composed of facilitated and structured steps, in broad agreement with current recommendations [8,9]. The members of the board initially agreed on areas of interest (ideas) through an NGT session held on 16 July 2020.

The overall process was divided into the following steps. First, each member of the board independently produced ideas, expressed in short sentences, which were deemed to be of interest. At this stage there is no limit to the ideas that each participant can indicate. A list of 46 statements was then produced with no discussion. A senior epidemiologist (GP), trained in gaining consensus among stakeholders (facilitator), then reorganized and categorized the ideas, which were then discussed on voted upon independently and a priority was assigned. Based on priority, a list of 14 topics were chosen.

**Figure 1:**
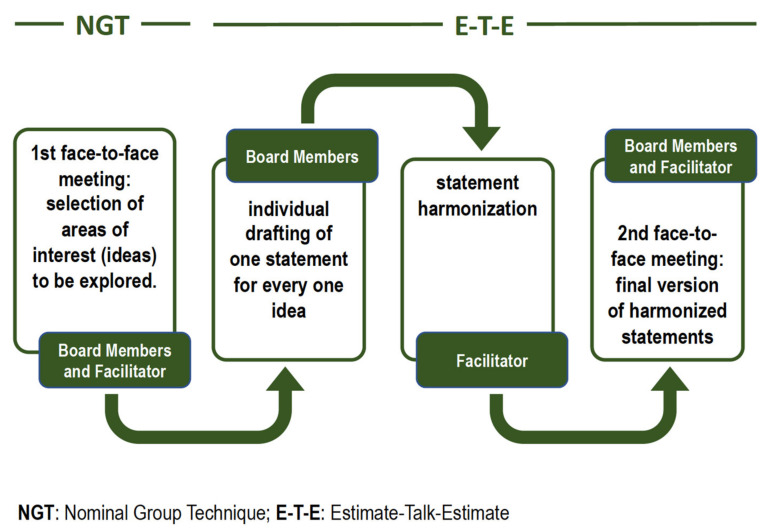
Overall process used to obtain consensus.

Afterward, finalized topics were used by board members to draft one statement for each idea individually through an Estimate–Talk–Estimate (E–T–E) approach [10,11]. This process resulted in a certain number of statements, which were then harmonized by the facilitator. The E–T–E, similarly to NGT combines a nominal group activity restricting verbal interaction with face-to-face interaction processes [12]. In the second face-to-face meeting, the board members and the facilitator reviewed and further discussed the harmonized statements, reaching a final version. The overall process is summarized in Figure 1.

## 3. Results

A total of 14 statements were drafted, discussed, modified, and approved by the board of experts (Table 1). Each of the statements is commented upon below along with the main supporting evidence.

### 3.1. Multidisciplinary Management

Multidisciplinary care of patients with NENs at referral centers has been associated with improvements in diagnosis, planning of treatment, and overall survival, as well as greater satisfaction by both the patient and clinician [13]. The role of a multidisciplinary team (MDT), which plays a pivotal function in the care of patients with NENs, should be always promoted in order to share common indications, optimize therapeutic strategies and allow integration of treatments, also between different centers. Considering these aspects, the participants agreed and strongly suggested that a network among “tumor boards” (dedicated to patients with NENs) is advisable. Adopting a similar approach independently of local experience, the harmonization of diagnostic and therapeutic pathways may be obtained everywhere. In addition, patients treated at two or more institutions can become part of an integrated therapeutic program generated from the cooperation among specialists from the different centers involved.

### 3.2. Baseline Prognostic Characterization

**Figure 2:**
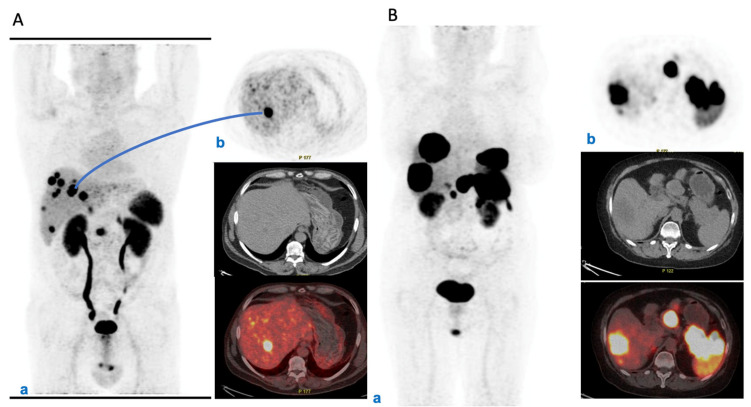
(**A**) PET/CT initial staging in metastatic pNET. (a) Male, 62 years old, pNEN, G1, initial staging. PET/CT ^68^Ga-DOTATOC MIP: depicts the intense uptake within primary pancreatic NEN (SUVmax 16.6) and in multiple liver metastases (SUVmax range: 6.6-62). (b) Axial image of the hottest liver metastasis along with corresponding CT and fused slice. (**B**) Female, 68 years old, pNEN, G3, staging during therapy with SSA and FOLFIRI. PET/CT ^68^Ga-DOTATOC MIP: (a) depicts the intense uptake within primary pancreatic NEN (SUVmax 38.6) and in large liver metastases (SUVmax range: 3-92). (b) Axial image of the primary pancreatic NEN, mesenteric lymph node, and largest liver metastasis.

The panel agreed that initial prognostic characterization should be based on clinical information as well as histopathological analysis and morphological and functional imaging. In advanced disease, for all NETs somatostatin receptor (SSTR) imaging with ^68^Ga-SSAs PET/CT has a main role in this context, and can combine prognostic, staging, and predictive information [14,15,16,17] (Figure 2A,B).

Moreover, the sensitivity and specificity of ^68^Ga-SSAs PET/CT for most NETs is high (>90%), except for insulinomas, which express SSTRs less frequently [18]. ^68^Ga-SSAs PET/CT can be useful in guiding the therapeutic strategy, as patients with a high and homogeneous expression of SSTRs are selected for radiolabeled SSAs [19]. ^18^F-FDG PET can be useful for NECs and NETs with high Ki-67, but also for NETs with low or inhomogeneous expression of SSTRs. Elevated ^18^F-FDG uptake, is a negative prognostic factor [20,21]. There is insufficient evidence for the use of circulating chromogranin A as a routine prognostic marker [22].

For resected NENs, a number of pathological factors have been associated with prognosis, such as tumor stage (pTNM), tumor grade, tumor size, and vascular/lymphatic/perineural invasion, and there are several nomograms that can be used to classify the patient’s risk of disease recurrence or progression [23,24,25].

Tumor tissue samples, preferably histological, should be always obtained (by percutaneous biopsy or surgery) for diagnosis and classification before starting medical anti-cancer treatment [26]. Endoscopic ultrasound-guided fine-needle biopsy (EUS-FNB) is crucial for the evaluation of pancreatic neuroendocrine tumors [27]. In addition to tumor differentiation (well, moderately, and poorly), the grade should be determined using the Ki-67 and mitotic index. The Ki-67 proliferative index is the most commonly used prognostic factor [28,29] and should be requested to pathologists, if not present in the initial report.

### 3.3. Watchful Waiting

A watchful waiting strategy means clinical observation to assess the spontaneous clinical history of the tumor in the absence of anti-tumor therapy [30]. Furthermore, its application in clinical practice can differ in terms of type and timing of imaging or other exams utilized [30]. In locally advanced/metastatic NENs, the experts did not recommend a watchful waiting strategy. Although watchful waiting has been reported in several guidelines and recommendations, it has never been validated, nor has it been specifically investigated or standardized. In patients with metastatic NENs, watchful waiting does not seem to have a role, on the basis of the results from the PROMID and CLARINET trials [31,32]. Watchful waiting to delay first-line therapy for a short period may be justified in asymptomatic patients with good performance status and a low-grade NET with the aim to better characterize the disease and define the optimal therapeutic strategy [14,33,34]. However, in metastatic disease a *watchful waiting* to definitively avoid treatment is not justified, as even NENs with very favorable biological characteristics tend to grow [30].

### 3.4. Follow-Up of Radically Resected NENs

Follow-up has been recommended in virtually all patients after radical resection of local or locally advanced and metastatic NENs [35,36]. Generally, guidelines and recommendations suggest that following complete resection morphological imaging is recommended every 3–6 months for 5 years and then every 12–24 months for up to 10 years [35,37]. The expert panel of this consensus suggests that, considering the long-term nature of follow-up, magnetic resonance imaging (MRI) with diffusion-weighted (DW) sequences should progressively substitute and has to be preferred over computed tomography (CT) with the aim of reducing exposure of the entire body to ionizing radiation and renal exposure to iodinated contrast media. Nevertheless, the choice of the morphologic modality has to be based on its accuracy in the visualization of the target lesions. Of note, periodic functional imaging (namely ^68^Ga-SSAs PET/CT) has not been demonstrated to have clinical utility in radically resected NETs, and is recommended only in patients with suspected recurrence of disease at morphological imaging or in those presenting new, suspicious, clinical signs and/or symptoms [14,15,33,35]. In general, considering that NENs are remarkably heterogeneous and this heterogeneity greatly influences the risk of relapse or progression and patient prognosis, the expert panel agreed that a fixed follow-up schedule might be inadequate in many cases. Current guidelines do not mention the possibility of risk-adapted individual follow-up. The experts agreed that follow-up for radically resected NEN should be patient/tumor tailored; the timing should be based on individual prognostic parameters, with a balanced analysis of risks and benefits. Stratification by risk of recurrence can help the clinician in avoiding unnecessary examinations in low-risk patients (e.g., reduction of exposure to radiation). In this regard, there is some evidence to suggest that the frequency of follow-up investigations can be based on tumor features, such as tumor differentiation, Ki-67 index, presence of metastases, and tumor size, even if no formal consensus has been reached in this regard [23,25]. Due to the well-known heterogeneity of NENs, it is clear that follow-up cannot be standardized on the basis of the primary site or pathology classification only, e.g., the WHO. It should be contextualized based on the specific characteristics of the disease in the individual patient and discussed within the NEN-dedicated multidisciplinary team. In other words, follow-up should be personalized.

### 3.5. Therapeutic Strategies

The expert panel recognized that there is little consensus on the optimal sequence of treatments for patients with NENs [7]. Patients with G1- and low G2 NETs that are not amenable to surgery often receive SSAs as first-line therapy [6,14,35], as recommended by international guidelines [7,38], in order to control tumor growth and/or associated clinical syndromes. For patients who show tumor progression after first-line treatment with SSAs, the selection of second-line therapy may be difficult due to the lack of an absolute standard. The sequence SSAs followed by PRRT/RLT upon progression has become a common/standard approach in G1-G2 SI-NEN patients, thanks to the results of the NETTER-1 trial [39]. The panel agreed with a previous suggestion that comorbidities and goal of treatment can help to drive the therapeutic choice [14]. For example, if the goal of therapy is to achieve tumor shrinkage, then various treatments, mainly chemotherapy and PRRT, may be considered according to the evidence, to be discussed within the NEN-dedicated MDT [40]. In a selected patient population and after a careful multidisciplinary discussion, a cytoreductive surgery on primary malignancy could be considered, due to the potential positive relationship of this approach with patient survival in retrospective case series [41,42].

If, however, effective long-term control of the endocrine clinical syndrome is the priority, then the most appropriate targeted therapy must be chosen. Patients with a malignant pancreatic insulinoma, for example, can gain long-term blood glucose control with everolimus, which would thus be preferred to control clinical progression vs. a SSA. Everolimus could even be continued in order to control the syndrome even in case of further progression, in association with subsequent tanti-tumor therapies (e.g., chemotherapy or PRRT), at least for a short period [43]. Conversely, based on its effects on glucose metabolism, sunitinib could have detrimental effects in patients with an insulinoma [44], and might be preferentially indicated in patients with a glucagonoma.

Systemic therapies can be suitably integrated with loco-regional therapies when clinically indicated and following multidisciplinary discussion. Liver-directed treatments (LDTs) such as trans-arterial chemoembolization (TACE), trans-arterial embolization (TAE), and thermo-ablation (TA), in fact, are usually considered for selected patients with liver metastases from NETs [6,45]. Finally, when deciding the sequence of treatments, additional toxicities should be taken into consideration as well as their impact on the patient’s quality of life.

### 3.6. Informed Consent for RLT

The expert panel agreed that specific and detailed, oral and written information should be given to the patients before obtaining the signed consent form before starting treatment. The information provided should include notes about the purpose, procedure, and risk-benefit balance deriving from radiation use in RLT. Moreover, the potential for early and late side effects (reversible hematological toxicity, nephrotoxicity), and the rare but severe long-term complications (myelodysplastic syndromes (MDS) and leukemia) have to be exhaustively and comprehensively discussed with the patient [43,46]. A relevant item concerns the information for patients (of both sexes) about the period of abstention from procreation.

### 3.7. Dosimetry of RLT

Dosimetric evaluation is currently not recommended during standard RLT, since the NETTER 1 trial demonstrated that four fixed doses of ^177^Lu- Lutathera^®^ (Basel, Switzerland, Novartis)(7.4 GBq) in most patients are characterized by a favorable toxicity profile and are effective [47]. Dosimetry should optimize the efficacy of therapy and minimize potential side effects to the organs at risk, namely red bone marrow and kidney. The use of individual dosimetry during RLT has the potential to tailor treatment after the standard four cycles [46], possibly receiving additional cycles (up to 10) before reaching dose-limiting toxicity levels [48,49].

In a prospective study with dosimetric assessment, patients who had, after the 4 cycles, an absorbed dose to the kidneys ≥23 Gy showed significantly better survival outcomes than those who did not reach such a preset dose [50]. Thus, using a predetermined cut-off of four cycles of 7.4 GBq ^177^Lu-DOTATATE, some patients would benefit from additional therapy, further highlighting the value of dosimetric evaluation. The panelists suggest that dosimetry should be performed in trials or for re-treatment. In this setting, the development of more accurate, simplified, and standardized methods will enable routine use of dosimetry in a clinical setting.

### 3.8. Management of Patients with Comorbidities

Comorbidities and safety of medical therapies must be always considered when choosing the most appropriate treatment. Comorbidities not representing an absolute contraindication to RLT (i.e., severe hypertension, brittle diabetes, functioning tumors, concomitant meningioma, etc.) should require specific protocols. Eligibility for RLT requires the absence of a significant impairment of renal function (creatinine clearance <30 mL/min). Given that some comorbidities are related with a higher risk of adverse reactions [51], patients with the certain characteristics should be more strictly monitored during treatment and considered for dose reduction or postponement of therapy. These include morphological abnormalities in the kidney/urinary tract, incontinence, creatinine clearance 30–50 mL/min, prior chemotherapy, diabetes mellitus, hypertension, heart failure, pre-existing hematologic toxicity (other than lymphopenia) ≥ grade 2 prior to therapy, and widespread bone metastases, as well as previous radiometabolic therapies, (including radioiodine therapy previously performed for thyroid cancer) and extended external bean radiation modalities.

In terms of medical therapies, sunitinib should be preferred over everolimus for patients with a pre-existing diabetes mellitus or underlying pulmonary disease, whereas everolimus should be preferred over sunitinib in patients with cardiovascular diseases, arterial hypertension, or bleeding diathesis [14]. In patients with mild and moderate hepatic impairment (Child-Pugh A-B), the dose of everolimus should be reduced down to 5 mg/day, respectively [6].

### 3.9. Management of Therapy with SSA during RLT

The association of SSAs and RLT has been suggested to play a role in tumor growth control [52]. A recent retrospective study reported better survival for patients with advanced NENs receiving combined treatment with SSAs and RLT vs. RLT alone [52]. While the type of SSA and its formulation and dose are yet to be standardized, the experts held that SSA therapy should be continued during the entire course of RLT, with dose adjustment in patients with functioning tumors.

In the NETTER-1 study, the combination of ^177^Lu-DOTATATE and octreotide LAR 30 mg every 4 weeks was reported to be safe with longer progression-free survival (PFS) and higher overall response rate (ORR) compared to high-dose octreotide LAR alone [47]. The PRELUDE study further demonstrated that the combination of lanreotide and ^177^Lu-DOTATOC/DOTATATE was effective and safe in patients with metastatic or locally advanced NENs [53]. Thus, the available evidence appears to suggest that the association of either octreotide or lanreotide with RLT is both safe and feasible, even if further studies are advisable.

### 3.10. Evaluation of Tumor Response (Morphological vs. Functional and Clinical) after RLT

**Figure 3:**
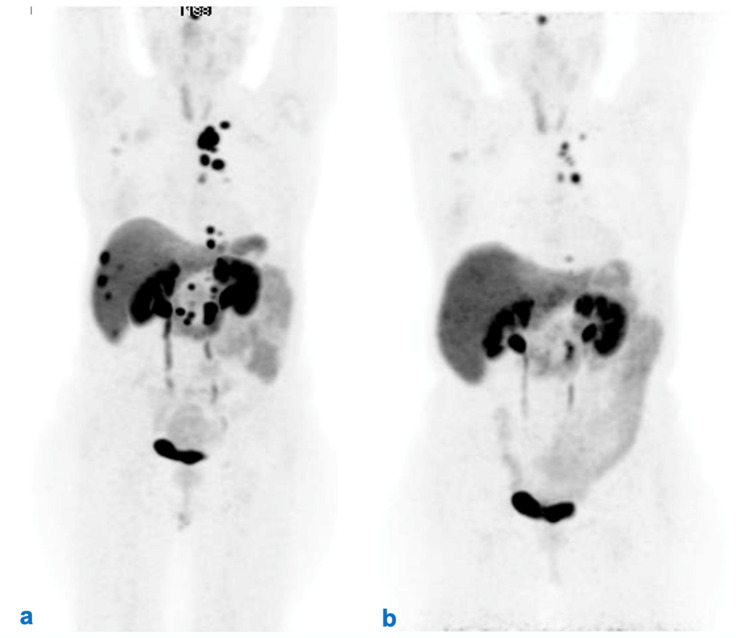
Monitoring response to RLT with PET/CT ^68^Ga-DOTATOC. Female, 58 years old, pNEN, G2, surgically removed in 2016. Staging before and after RLT with ^177^Lu-Lutathera. PET/CT 68Ga-DOTATOC MIP (**a**) depicts the extent metastatic disease (thoracic, axillary and abdominal LNs, liver metastases) before RLT. (**b**) MIP after RLT with no evidence of liver metastases and abdominal lymph nodes along with a significant reduction in the radioligand uptake of thoracic lymph nodes, which is suggestive for a partial response.

The expert panel held that tumor response assessment after RLT should carefully consider both morphological and functional imaging, and that the timing of imaging should be correlated with characteristics of the individual tumor based on histopathological, morphological, functional, and clinical parameters. Evaluation of morphological tumor response (with CT-scan and/or MRI) is mandatory in all patients undergoing medical therapies of a NEN and is usually based on RECIST 1.1 criteria [35,37]. Moreover, radiological tumor response assessment should be made comparing the same technique (e.g., CT-scan or MRI). The preferred imaging modality should be chosen initially on an individualized basis depending on how well it allows visualization of the parameter tumor lesions at baseline [14,35,54]. In this sense, PET with FDG could also be useful in evaluating the response (Figure 3).

Functional imaging also plays a role in evaluation of response to RLT. Appearance of new uptake lesions and/or disappearance of previous uptake areas at ^68^Ga DOTA-peptide PET/CT may mean tumor progression or regression [54,55]. A decrease in uptake at ^68^Ga DOTA-peptide PET/CT after RLT may be a predictor of longer PFS and improvement of symptoms [56]. Conversely, loss of SSTR expression at ^68^Ga DOTA-peptide PET/CT and the appearance of 18F-FDG uptake on the same or different lesions may be associated with rapid tumor progression and poor prognosis [57,58].

### 3.11. Follow-Up after RLT

As with the prior statement, follow-up should be patient-tailored and include both morphological (CT and/or MRI) and/or functional (PET/CT with radiolabeled somatostatin analogs and/or ^18^F-FDG) imaging and biomarkers chosen based on the characteristics of the tumor. The timing should be based on the prognosis, avoiding unnecessary use. Additional use of imaging modalities is justified when discordant results are obtained by CT (i.e., stable lesions) and PET/CT (increased uptake or greater number of detected lesions); the suggestion might be to repeat the PET/CT in 2 or 3 months to verify the extent of the disease. The most appropriate use of morphological and functional imaging modalities should also be guided to minimize the doses of ionizing radiations to patients considering the prospect of long-term follow-up. Following RLT with ^177^Lu-DOTA-SSAs, clinicians should be aware of previously-identified predictors of poor outcomes which can help to stratify patients by risk [59]. These include high hepatic tumor load and skeletal metastases, elevated blood chromogranin A, metastases at uncommon sites, and ascites [59].

### 3.12. Off-Label Use of RLT

While acknowledging that off-label use of RLT is possible, it was held that alternative schedules, types of administration, indications other than those approved, and rechallenge should be limited to specific clinical studies.

A standard course of ^177^Lu-DOTATATE RLT consists of four cycles administered every 6–8 weeks [57]. It is believed that optimal results are achieved when the dose absorbed is close to, but not exceeding, the maximum acceptable dose for radiosensitive organs [50]. Given this, by relying on individual dosimetry, a substantial proportion of patients could possibly receive additional cycles of RLT before reaching dose-limiting toxicity for the kidneys and bone marrow [49]. However, it should be considered that the relationships between the dose absorbed and the clinical effects depends on unknown factors such as dose rate, intracellular distribution of the radionuclide, and radiosensitivity of the tumor. Additional data are needed to clarify the precise role of RLT beyond a standard course.

While the combination of ^90^Y-DOTATOC and ^177^Lu-DOTATATE has been advocated by several groups [60,61], and some studies have documented a higher ORR and survival advantages using the combination [62,63], in the opinion of the expert panelists these regimens cannot yet be recommended in routine clinical practice in the absence of additional information about their safety and efficacy.

In selected patients who initially respond to RLT, but subsequently progress, retreatment with RLT might be considered up to a lifetime maximum of around eight cycles [64,65,66]. Indeed, salvage therapy with ^177^Lu-DOTATATE has been documented to be both safe and effective even in patients who underwent prior, extensive multimodal treatments [67,68,69]. In a phase II trial investigating retreatment with low-dosage ^177^Lu-DOTATATE in 26 patients who progressed after ≥12 months following ^90^Y- DOTATOC reported a disease control rate of 85%, indicating that in some patients retreatment with RLT may be a valid therapeutic option for progressive disease [70]. Data on the efficacy of RLT retreatment in patients with advanced NET are depicted in Table 2 [67,68,69,70,71,72].

Since RLT is often associated with good responses and is generally well tolerated, this has stimulated its use beyond the indicated recommendations. Such a situation includes G3 NENs with a Ki-67 index between 20% and 30% [7,73,74]. Since high ^18^F-FDG uptake is generally observed in these patients, combined chemo-RLT may be a reasonable therapeutic option [75].

**Figure 4:**
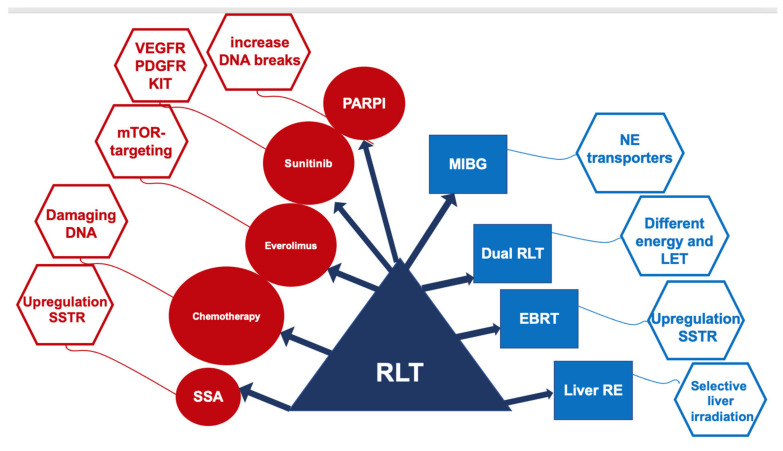
Current therapies proposed in combination with RLT.

There is some encouraging evidence suggesting that RLT efficacy could be improved by the concomitant administration of several antineoplastic therapies (Figure 4).

Treatment with SSAs can upregulate SSTR. The overexpression of the tumor targets SSTR2 in NETs can increase the effectiveness of RLT without increasing the toxicity profile. More than one-third of patients with progressive NETs in the multicenter retrospective trial PRELUDE, treated with SSA lanreotide combined with RLT, had an objective response, and 95% were, at the last follow-up visit 1 year post-treatment, still progression-free [53].

In patients with NETs characterized by heterogeneous grading, with lesions simultaneously showing high and low Ki-67 values, the combined use of RLT and chemotherapeutic regimen with capecitabine and temozolomide (CAPTEM) has been reported to be effective. However, such a combination is suggested to be adopted in dedicated protocols taking into account the potential toxicity of CAPTEM in combination with RLT [76,77,78].

Clinical experience with the combined treatment of everolimus and RLT is extremely limited. In a phase I study, patients received escalating doses of everolimus: 5 to 10 mg/d for 24 weeks, and RLT, the maximum tolerated dose of everolimus in combination with RLT was 7.5 mg/d [79].

An ongoing randomized phase II study is aiming to compare the efficacy of sunitinib and RLT in advanced metastatic pancreatic NETs (NCT02230176). The focus is to determine the results of the cross-over groups, since sunitinib seems to be a potential radiosensitizer that might improve the effects of RLT. However, to date, there are no substantial clinical data on the combined use of RLT and sunitinib.

Combination of the anti-PD-1 checkpoint inhibitor nivolumab with increasing doses of RLT has been tested in a phase I/II trial including nine patients with small cell lung cancer (NCT03325816). Low-level activity RLT (3.7 GBq LUTATHERA) every 8 weeks and nivolumab every 2 weeks for a period of 6 months showed no dose limiting toxicity. More intense RLT (7.4 GBq LUTATHERA) led, in the six patients with measurable disease, to one partial response and two stable disease, with a single case of a grade 3 rash [80].

Another promising partner of RLT might be poly (ADP-ribose) polymerase-1 inhibitors (PARPi). In preclinical studies, PARPi combined with PRRT increased DNA double-strand tumor breaks and increased survival compared to PRRT as a monotherapy [81].

A recent sub-analysis of the NETTER-1-study showed that PFS in NET patients with large tumor lesions (>3 cm in diameter) was significantly shorter (*p* = 0.022) than in patients with small lesions [82]. A possible explanation for the failure in large liver lesions is due to the maximum tissue penetration of ^177^Lu, which is limited to 2–4 mm. In a comparative analysis, patients treated with radioembolization plus RLT showed a superior OS (87% vs. 67%) than those receiving radioembolization alone (68 months vs. 35 months) [83]. Radioembolization after initial RLT is feasible, with objective responses of 16% after ^90^Y and 43% after ^166^Ho radioembolization. Such combined therapies should be verified in larger cohorts of patients with prevalent liver spreading of NETs, also focusing on the related hepatotoxicity, which may lead, besides the radionuclide, used to death [83,84].

Tandem RLT (using ^177^Lu- and ^90^Y-DOTA-SSA), in published series, shows a better overall survival than RLT with 90Y-DOTA-SSA alone (5.51 y vs. 3.96 y) along with a higher response rate and similar related toxicity [85,86]. At present, the off-label use of RLT should be limited to specific clinical circumstances and should always be discussed within the NEN-dedicated MDT. These studies are summarized in Table 3.

### 3.13. Approach to Patients with Bone Metastases

In this statement, the experts recommended that bone involvement detected by appropriate imaging techniques must be carefully assessed in patients with a metastatic NEN to identify patients at risk of skeletal-related events (SREs). Bone metastases are detectable in 10–20% of patients with NENs and associated with poor prognosis [93]. Bone metastases are usually identified using appropriately sensitive functional imaging techniques, such as ^68^Ga-DOTA-peptide PET/CT [94,95]. Bone MRI can also be performed to assess suspicious lesions [7,35]. At present, it remains uncertain if identification of micro-metastases (<5 mm) to bone should prompt to changes in management [35]. Palliative radiotherapy should be considered for patients with painful bone metastases that are difficult to control with medical therapy and for bone lesions at sites with a high risk of clinical complications [93,96]. Relief of pain has been described in the majority of patients treated with external beam radiotherapy [96]. In a prophylactic setting, radiotherapy may be beneficial in avoiding bone fractures [97]. Surgical therapy for neuroendocrine bone metastases is rarely indicated and mostly for mechanical reasons or isolated lesions [93]. Although there is little practical guidance, bisphosphonates or rank ligand inhibitors can be administered [97,98].

When required for disease control, symptomatic patients with bone metastases generally require systemic chemotherapy [93]. However, the optimal regimens are still debated and are likely to depend on the site of the primary tumor. RLT may be effective in some patients with bone metastases, who demonstrated high expression of SSTRs. In fact, two retrospective series have shown that RLT appears to be associated with ORR in bone lesions in around half of patients with NENs and bone metastases, although there is a potentially increased risk of myelotoxicity [99,100]. However, additional studies are warranted to confirm this data.

### 3.14. Role of Patient-Reported Outcomes in Management

Increasing importance is being given to patient-reported outcomes (PROs) in many fields of oncology, which are used to evaluate the health and quality of life of patients. The FDA defined PROs as “any report of the status of a patient’s health condition that comes directly from the patient, without interpretation of the patient’s response by a clinician or anyone else” [101]. Even in clinical trials, the use of PROs has become common in patients with NENs [102,103,104]. PROs allow for integration of clinical outcomes with the patient’s opinion of their own health [105]. This is important since NENs pose considerable burden for patients [105]. PROs should be incorporated in oncology to guarantee optimal delivery of patient-centered care. Furthermore, the routine evaluation of PROs will allow clinicians to better recognize and understand the unmet needs of patients with NENs. PROs can be evaluated using validated tools such as the EORTC QOL-C30 questionnaire and, in the opinion of the experts, should receive greater consideration by management guidelines in the future, which is at the basis of this statement.

## 4. Conclusions

Herein, consensus on a series of statements regarding diagnosis and clinical management of patients with NENs was reached by a panel of experts. The statements covered a broad range of topics from tools for diagnosis to follow-up, evaluation of response, treatment efficacy, therapeutic sequence, and watchful waiting. Most of these topics are not addressed directly in treatment guidelines, and in the opinion of the board members additional guidance would thus be helpful in daily practice. The experts tried to define indications and suggestions, taken from the existing literature and their own experience.

At present, RLT is both effective and safe for a large proportion of patients. Therefore, it is crucial to optimize RLT for NET patients starting from accurate characterization of the patient and his/her disease. This initial characterization must be based on clinical information as well as histopathological analysis, morphological and functional imaging useful in guiding the therapeutic strategy. Somatostatin receptor imaging with ^68^Ga-SSAs PET/CT has a main role for selecting patients who can be treated with radiolabeled SSAs. In our opinion, the future challenges for RLT involve not only the optimal therapeutic advantage by focusing on more precise dosimetric protocols, but also in greater understanding of the genotypic and phenotypic characteristics that differentiate the various subpopulations of NET patients [106]. Only in this way will it be possible to identify and stratify the potentially “responsive” and “non-responsive” forms to RLT [107]. The result will be the earlier and more accurate selection of patients, who can avoid ineffective treatments with unnecessary toxicity and benefit from the most appropriate line of therapy, with increased expectations and quality of life. This methodological approach can also bring about the definition of shared guidelines and standardized therapeutic algorithms that can aid in unravelling the biological, clinical, and prognostic uncertainties that still surround NENs. RLT with ^177^Lu-DOTATATE is a well-established second-line treatment, after SSA, of SI-NENs G1 and G2, approved by EMA and FDA [47]. For pancreatic NENs, there is no similar evidence, lacking head-to-head comparisons with everolimus or sunitinib. However, RLT may have greater efficacy with better safety compared to the two targeted therapies. The experts did not exclude the opportunity to consider RLT as second line therapy in all GEP NETs (G1 and G2) with a strong and homogeneous expression of SSTR at ^68^Ga-PET/CT, always considering comorbidities, goals of treatment, and treatment-related adverse events as well as the patient’s QoL. Radioligand therapy may also be effective (ORR) in some patients with bone metastases with high expression of SSTRs.

Follow-up should be patient/tumor tailored with a shared plan about timing and type of imaging procedures to use in order to avoid safety issues. Stratification of patients, by risk of recurrence based on individual prognostic parameters and tumor features, can help clinicians in avoiding unnecessary and potentially invasive examinations. Dosimetry evaluation is recommended to optimize the efficacy of RLT and to minimize dose limits exceeding for the organs at risk. The use of dosimetry during RLT has also the potential to safely administrate supplementary cycles that may be associated with better survival outcomes indicating that in some patients’ retreatment may be a valid therapeutic option for progressive disease.

The experts also stressed that PROs should receive greater attention during treatment and follow-up, given that they provide important insights to treating physicians about the patient’s perspective. Another important aspect is the role that the NEN-dedicated MDT should have in NEN patient care. A multidisciplinary approach should be mandatory, and whenever feasible within the context of a NEN-referral center. The MDT should be dedicated to NEN, in the sense that each specialist should have particular expertise in NEN field and routinely interact with colleagues from different specialists deeply involved in NEN. In this regard, and in order to achieve greater harmonization in treatment and facilitate comparison among centers and therapies, a series of quality indicators have been recommended for care of patients with NENs, which include the use of a detailed pathology report and tumor board review was also included among the performance indicators [108]. In considering harmonization of care, the therapeutic benefits of RLT should be considered while at the same time minimizing the use of off-label RLT and watchful waiting unless carried out within part of a dedicated clinical study. While several aspects in the treatment of NENs undoubtedly warrant additional study before specific recommendations can be made, clinicians should obviously use evidence-based best judgment according to the individual characteristics of the patient and tumor, as well as regulatory aspects. Due to the clinical heterogeneity and the relative lack of absolute evidence in NENs, personalization of the diagnostic–therapeutic work-up is crucial, more than in other fields of oncology.

## Figures and Tables

**Table 1:** Statements on diagnosis and management of NENs.

	Statement
1. Multidisciplinary discussion	A network among “tumor boards” working on NEN patients is advisable NEN-dedicated multidisciplinary teams should adopt the same main criteria independently of local experience.
2. Initial prognostic characterization	Initial prognostic characterization should be based on clinical information (functioning/non-functioning, performance status, comorbidity), histopathology (differentiation and grading), and morphological and functional imaging. There is no recommended definition of disease at high risk after radical surgery across NEN primary diseases.
3. Watchful waiting	A watchful waiting strategy is generally not recommended in locally advanced/metastatic patients.
4. Follow-up of radically resected NENs	Follow-up should be patient-tailored in patients with NEN after radical surgery and should include a panel of conventional tests, including circulating markers, plus a list of optional instrumental tests, chosen based on the characteristics of the tumor and patient. A patient-tailored long term follow-up strategy is still lacking and needs to be defined. The timing should be modulated on the basis of prognostic parameters, while strongly taking into account safety issues related to potentially invasive exams.
5. Therapeutic strategies	There is poor evidence regarding a specific sequence or integration of various treatments in NENs. The therapeutic strategy with sequence and type of treatments should be decided in a tumor board considering the characteristics of the patient, literature data, and regulatory aspects.
6. Informed consent for RLT	A standard informed consent form for RLT should be used. Informed consent should include specific information about the purpose, mode of execution, risk-benefit balance, and potential for early and late side effects, allowing optimization of communication about the risks, benefits, and possible alternative options, to provide the same level of information within all institutions.
7. Dosimetry of RLT (for therapy)	Dosimetry evaluation should be recommended to prevent potential risks to bone marrow and kidney function to provide data to clinicians, especially in patients with long survival expectancy.
8. Management of patients with comorbidities	Comorbidities not representing an absolute contraindication to RLT (i.e., severe hypertension, brittle diabetes, functioning tumors, concomitant meningioma, etc.) should require specific protocols.
9. Management of therapy with SSA during RLT	SSA therapy should be continued during the entire course of RLT. Dosage may be adjusted in case of functioning tumors.
10. Evaluation of response (morphological vs. functional and clinical) after RLT	Assessment of tumor response after RLT should carefully consider both morphological and functional imaging. However, the timing of imaging should be correlated with characteristics of the individual tumor.
11. Follow-up after RLT	Follow-up should be patient-tailored and include morphological (CT and/or MRI) and/or functional (PET/CT with radiolabeled somatostatin analogs and/or FDG) imaging and biomarkers, chosen based on the characteristics of the tumor. The timing should be modulated based on prognostic parameters, while strongly considering safety issues. It is suggested to intercalate morphological and functional imaging to reduce the patient’s irradiation dose given the very long follow-up.
12. Off-label use of RLT	Alternative schedules, means of administration, indications other than approved, and rechallenge should be limited to specific clinical studies.
13. Approach to patients with bone metastases	Bone involvement with appropriate imaging techniques must be carefully assessed in patients with a metastatic NEN to identify those at risk of skeletal-related events.
14. Role of PROs in management	Patient-reported outcomes (PROs) should be considered as a critical endpoint of benefit. Thus, guidelines should consider PROs, pointing out that their lack may have a bearing on the ultimate recommendation.

**Table 2:** Efficacy of radioligand therapy re-treatment in patients with advanced NET.

Study	Number of Patients	Initial RLT	Re-Treatment RLT	PFS (Months)	95% CI
Sabet et al., 2014 [72]	33	^177^Lu-DOTATATE	^177^Lu-DOTATATE	13.0	9.0–18.0
Severi et al., 2015 [70]	26	^90^Y-DOTATOC	^177^Lu-DOTATATE	9.0	5.0–17.0
Vaughan et al., 2018 [69]	47	^177^Lu-DOTATATE or 90Y-DOTATOC	^177^Lu-DOTATATE or 90Y-DOTATOC	17.5	11.0–23.8
Baum et al. [71]	470	^177^Lu-DOTATATE or ^90^Y-DOTATOC	^177^Lu-DOTATATE or ^90^Y-DOTATOC	11.0	9.4–12.5
Van der Zwal et al., 2019 [68]	168	1^77^Lu-DOTATATE	^177^Lu-DOTATATE	14.6	12.4–19.6
Rudisile S et al., 2019 [67]	32	^177^Lu-DOTATATE	^177^Lu-DOTATATE	6.0	0.0–16.00

**Table 3:** Efficacy and safety of combination treatment with RLT.

Combination Partner	ORR (%)	OS (Months)	PFS (Months)	SAE (%)	Reference
SSA	37	NR	48	3% hepatoxicity	[52,53,65]
Capecitabine	24–30	NR	31	15% hematotoxicity	[87,88]
CAPTEM	53–70	NR	22–48	6% hematotoxicity	[76,77,78]
5-FU	25	NR	-	-	[89]
Everolimus	44	NR	63 at 2 years	100% hematotoxicity	[79]
EBRT	0	NR	108	-	[90]
Liver Embolization					
(^90^Y)	16	42–68	-	50% liver enzyme elevation	[83,84,91]
(^166^Ho)	43		-	10% abdominal pain	
Dual RLT (^177^Lu/^90^Y)	42	66–127	-	2% MDS	[63,85,86]
(^177^Lu/^225^Ac)			-	7% hematotoxicity	
MIBG (^131^I)	0		-	33% thrombocytopenia	[92]
